# Associations of Dietary Glucose, Fructose, and Sucrose with β-Cell Function, Insulin Sensitivity, and Type 2 Diabetes in the Maastricht Study

**DOI:** 10.3390/nu9040380

**Published:** 2017-04-13

**Authors:** Louise J. C. J. den Biggelaar, Simone J. P. M. Eussen, Simone J. S. Sep, Andrea Mari, Ele Ferrannini, Martien C. J. M. van Dongen, Karlijn F. M. Denissen, Nicole E. G. Wijckmans, Miranda T. Schram, Carla J. van der Kallen, Annemarie Koster, Nicolaas Schaper, Ronald M. A. Henry, Coen D. A. Stehouwer, Pieter C. Dagnelie

**Affiliations:** 1 Department of Epidemiology, Maastricht University, Maastricht MD 6200, The Netherlands; simone.eussen@maastrichtuniversity.nl (S.J.P.M.E.); mcjm.vandongen@maastrichtuniversity.nl (M.C.J.M.v.D.); karlijn.denissen@maastrichtuniversity.nl (K.F.M.D.); nicole.wijckmans@maastrichtuniversity.nl (N.E.G.W.); dagnelie@maastrichtuniversity.nl (P.C.D.); 2 Cardiovascular Research Institute Maastricht (CARIM), Maastricht University, Maastricht MD 6200, The Netherlands; s.sep@adelante-zorggroep.nl (S.J.S.S.); m.schram@mumc.nl (M.T.S.); c.vanderkallen@maastrichtuniversity.nl (C.J.v.d.K.); n.schaper@mumc.nl (N.S.); rma.henry@mumc.nl (R.M.A.H.); cda.stehouwer@mumc.nl (C.D.A.S.); 3 Care and Public Health Research Institute (CAPHRI), Maastricht University, Maastricht MD 6200, The Netherlands; annemarie.koster@mumc.nl; 4 Department of Internal Medicine, Maastricht University Medical Centre, Maastricht MD 6200, The Netherlands; 5 C N R Institute of Neuroscience, Padova 56124, Italy; andrea.mari@cnr.it; 6 C N R Institute of Clinical Physiology, Pisa 56124, Italy; ferrannini@ifc.cnr.it; 7 Heart and Vascular Centre, Maastricht University Medical Centre, Maastricht MD 6200, The Netherlands; 8 Department of Social Medicine, Maastricht University, Maastricht MD 6200, The Netherlands

**Keywords:** beta-cell function, diet, fructose, glucose, insulin sensitivity, prediabetes, sucrose, type 2 diabetes

## Abstract

The associations of glucose, fructose, and sucrose intake with type 2 diabetes mellitus (T2DM) have been inconsistent. Furthermore, there is a lack of studies focusing on early markers of T2DM that provide insight into the process of T2DM progression: impaired pancreatic β-cell function (BCF) and insulin sensitivity. This study evaluated associations cross-sectionally in a population-based cohort consisting of 2818 individuals (mean ± SD age 59.7 ± 8.18, 49.5% male, *n* = 120 newly diagnosed T2DM). Glucose, fructose, and sucrose intake were assessed by a food frequency questionnaire. Glucose metabolism status, insulin sensitivity, and BCF were measured by a seven-points oral glucose tolerance test. Linear regression analysis revealed a positive association of glucose intake with insulin sensitivity in the fully adjusted model (standardized beta (95% CI) 0.07 (0.05, 0.14) SD for ≥23 g vs. <10 g of glucose). Fructose and sucrose intake were not associated with insulin sensitivity after full adjustments. In addition, no associations of dietary glucose, fructose, and sucrose with BCF were detected. In conclusion, higher intake of glucose, not fructose and sucrose, was associated with higher insulin sensitivity, independent of dietary fibre. No convincing evidence was found for associations of dietary glucose, fructose, and sucrose with BCF in this middle-aged population.

## 1. Introduction

Lifestyle modifications, including the adoption of a healthier diet, have been suggested to reduce type 2 diabetes mellitus (T2DM) risk by 40%–70% [1]. Previous studies on the association of dietary intake of total mono- and disaccharides with T2DM have shown conflicting results. These conflicting results might partly be explained by opposite associations of individual mono- and disaccharides with T2DM [2]. The most commonly consumed, and most frequently studied individual mono- and disaccharides are glucose, fructose, and sucrose [3]. In some studies, positive associations of glucose and fructose with prediabetes [4] and T2DM [2,5] have been observed, whereas others revealed inverse [6] or absent associations [7]. Regarding sucrose intake, some studies showed inverse associations with prediabetes [8] or T2DM [2,9], whereas others showed nonsignificant associations [5,6,10].

β-cell function (BCF) and insulin sensitivity deteriorate years before T2DM diagnosis [11]. To intervene early in the process of T2DM development and to increase insight in the putative effects of mono- and disaccharides on more detailed level of defects in glucose metabolism, it is crucial to study determinants of BCF and insulin sensitivity. Defects in the intracellular insulin signaling pathway result in decreased glucose uptake by insulin-sensitive tissues [12]. If insulin sensitivity decreases, pancreatic β-cells normally upregulate insulin secretion in order to maintain normal blood glucose levels [13,14]. However, when β-cell function is impaired, plasma glucose levels rise into the prediabetic and diabetic range [13,14].

Studies evaluating the effects of mono- and disaccharide intake on insulin sensitivity are scarce and have resulted in conflicting findings [15,16,17]. Furthermore, to the knowledge of this study’s authors, no earlier studies focused on non-fasting measures of insulin sensitivity in order to provide important insights into overall insulin resistance (hepatic and peripheral insulin resistance), while fasting indices reflect mainly hepatic insulin resistance [18]. The effects of mono- and disaccharide intake on BCF are unknown. Therefore, the aim of the current study was to investigate the individual associations of glucose, fructose, and sucrose intakes with BCF and insulin sensitivity as primary outcomes, and with prediabetes and newly diagnosed T2DM as secondary outcomes.

## 2. Methods 

### 2.1. The Maastricht Study Design and Population

This study used data from the Maastricht Study, an observational prospective population-based cohort study. Its rationale and methodology have been described previously [19]. In brief, the study focuses on the etiology, pathophysiology, complications, and comorbidities of T2DM and is characterized by an extensive phenotyping approach. All individuals aged between 40 and 75 years and living in the southern part of The Netherlands were eligible for participation. Participants were recruited through mass media campaigns and from the municipal registries and the regional Diabetes Patient Registry via mailings. The representation with the source population in the study region was monitored continuously and was aligned with postal codes [19]. Recruitment was stratified according to known T2DM status, with an oversampling of individuals with T2DM, for reasons of efficiency. The study was approved by the institutional medical ethical committee (NL31329.068.10) and the Minister of Health, Welfare, and Sports of The Netherlands (Permit 131088-105234-PG). All participants gave written informed consent.

The present report includes cross-sectional data from the first 3451 participants who completed the baseline survey between November 2010 and September 2013. The examinations of each participant were performed within a time window of three months. 

For the present analyses, individuals who had another type diabetes than type 2 (*n* = 41), suffered from cancer (*n* = 122), had not filled out a food frequency questionnaire (FFQ) (*n* = 154), or had reported implausible total energy intake (<800 or >4200 kcal/day for men and <500 or >3500 kcal/day for women, *n* = 63) were excluded [20]. Individuals with either a missing fasting or 120 min post glucose load blood sample, or with overall less than five OGTT blood sampling points (*n* = 464) were excluded from the main analyses (primary outcomes: BCF and insulin sensitivity). This resulted in a study population of 2607 individuals for the primary outcome measures BCF and insulin sensitivity. For the analyses with newly diagnosed T2DM as an outcome, this study additionally excluded individuals with previously diagnosed T2DM (*n* = 738). For the analyses with prediabetes as an outcome, individuals with T2DM were excluded (*n* = 858). This resulted in a study population of 2213 and 2333 individuals for the secondary outcome measures prediabetes and newly diagnosed T2DM, respectively.

### 2.2. Glucose Metabolism

To determine glucose metabolism status, all participants—except those who used insulin—underwent a standardized 2 h 75 g oral glucose tolerance test (OGTT) after an overnight fast. Venous blood samples were collected before and 15, 30, 45, 60, 90, and 120 min after oral glucose load. 

Plasma for the assessment of insulin and C-peptide levels was collected in ethylenediaminetetraacetic acid (EDTA) tubes, stored on ice, separated after centrifugation (3000× *g* for 15 min at 4 °C), and stored at −80 °C until the assays were performed. The time between collection and storage was <2 h. Insulin and C-peptide were measured in never-thawed plasma by use of a custom duplex array of MesoScale Discovery (MesoScale Discovery, Gaithersburg, MD, USA). In short, 96 well-plates, with capture antibodies against insulin and C-peptide patterned on distinct spots in the same well, were supplied by the manufacturer. Samples (10 µL/well), detection antibodies and read buffer for electrochemiluminescence were applied according to manufacturer’s instruction and plates were read using a SECTOR^®^ 2400 Imager. Detection ranges of the assay were 35–25,000 pg/mL for insulin and 70–50,000 pg/mL for C-peptide. Interassay coefficients of variation for insulin and C-peptide were 10.1% and 8.2%, respectively. Insulin and C-peptide values were converted from pg/mL to pmol/L using a molar mass of 5808 g for insulin and 3010 g for C-peptide. 

Plasma for the assessment of glucose was collected in sodium fluoride/potassium oxalate (NaF/KOx) tubes on ice. Fasting and 120-min-postload plasma glucose were measured in fresh samples with the enzymatic hexokinase method by use of two automatic analysers (i.e., the Beckman Synchron LX20 (Beckman Coulter Inc., Brea, CA, USA) for samples obtained between November 2010 and April 2012, and the Roche Cobas 8000 (Roche Diagnostics, Mannheim, Germany) for samples obtained thereafter). Plasma for the assessment of glucose at other time points during the OGTT was separated after centrifugation (3000× *g* for 15 min at 4 °C) and stored within 2 h at −80 °C until the assays were performed. Glucose was measured in these never-thawed samples with the enzymatic hexokinase method by use of the Roche Cobas 6000 (Roche Diagnostics, Mannheim, Germany). The Pearson correlation coefficient between fresh and frozen samples were 0.96 and 0.99, respectively, for fasting and 120-min-postload plasma glucose samples (*n* = 486 samples) in a quality control.

Glucose metabolism status was defined according to the WHO 2006 criteria into normal glucose metabolism (NGM), impaired fasting glucose (IFG), impaired glucose tolerance (IGT), and T2DM [21]. Individuals without type 1 diabetes mellitus on diabetes medication were classified as having T2DM [19]. 

#### 2.2.1. β-cell Function and Insulin Sensitivity

As BCF consists of multiple components, it cannot be described by a single measure. Therefore, we used three mathematical model-based parameters (β-cell glucose sensitivity, the potentiation factor ratio, and β-cell rate sensitivity) according to a previously described model [22], and two classic, relatively simple BCF-indices (C-peptidogenic index and the ratio of the C-peptide to glucose area under the curve) [22,23,24,25]. 

The mathematical model parameter “β-cell glucose sensitivity” is the slope of the glucose-insulin secretion dose-response function [22], and represents the dependence of insulin secretion on absolute glucose concentration at any time point during the OGTT. β-cell glucose sensitivity is a sensitive index to quantify β-cell dysfunction [13,26,27]. The dose-response relationship is modulated by β-cell potentiation, which accounts for higher insulin secretion during the descending phase of hyperglycaemia than during the ascending phase of an OGTT for the same glucose concentration. β-cell potentiation is set as a positive function of time and averages 1 during the OGTT. Therefore, it represents the relative potentiation of the insulin secretion response to glucose. The β-cell potentiation parameter used in the present analysis represents the ratio of the β-cell potentiation factor at the end of the 2-h OGTT relative to the β-cell potentiation factor at the start. “β-cell rate sensitivity” is a marker of early phase insulin release, and represents the dynamic dependence of insulin secretion on the rate of change in glucose concentration [22]. 

The simple BCF-indices C-peptidogenic index (ΔCP_30_/ΔG_30_) and the ratio of the C-peptide to glucose area under the curve (CP_AUC_/G_AUC_) were also calculated. The C-peptidogenic index (the equivalent of the insulinogenic index) reflects early phase insulin secretion and has a good ability to discriminate between NGM and (pre)T2DM (ROC AUCs ≥ 78%) [23]. The CP_AUC_/G_AUC_ is a measure of overall insulin secretion. 

The Matsuda index (10,000/√G_0_ × I_0_ × mean G × mean I) was used as a measure of insulin sensitivity [28].

#### 2.2.2. Dietary Intake

All participants completed a food frequency questionnaire (FFQ) prior to being informed about their glucose metabolism status (e.g., NGM, prediabetes, or T2DM) [20]. 

Habitual dietary intake over the past 12 months was estimated by a tailor-made FFQ developed using the Dutch national FFQ tool [29]. The FFQ consisted of twenty-three product groups capturing 253 food items. Briefly, for each food item, frequency of consumption (ranging from “never or less than once a month” to “every day”) and consumed amount were asked. Based on the FFQ data, daily intakes of the 23 main food product groups and nutrients were calculated (g/day), and subsequently daily nutrient intakes. The main food product groups were: 1. Bread (10 food items), 2. Savoury bread spreads (2 food items), 3. Cheese (9 food items), 4. Milk and milk products (45 food items), 5. Eggs (1 food item), 6. Cereals and cereal products (9 food items), 7. Soups (2 food items), 8, Potatoes (4 food items), 9. Vegetables (28 food items), 10. Legumes, (1 food item), 11. Meat, meat products and poultry (16 food items), 12. Fish (8 food items), 13. Soy and vegetarian products (7 food items), 14. Herbs and spices (3 food items), 15. Mixed dishes (9 food items), 16. Fats, oils, and savoury sauces (26 food items), 17. Fruits (20 food items), 18. Sugar, sweets, and sweet sauces (11 food items), 19. Nuts, seeds, and snacks (7 food items), 20. Pastry and biscuits (13 food items), 21. Alcoholic and non-alcoholic beverages (26 food items), 22. Clinical formulas (1 food item), and 23. Miscellaneous foods (1 food item).

Intakes of total energy and individual mono- and disaccharides (glucose, fructose, and sucrose) were calculated by using the Dutch Food Composition Database (NEVO) of 2011 [30]. Missing values for mono- and disaccharide contents of specific products in the NEVO database were substituted with values obtained from other relevant food composition tables [31]: first, McCance and Widdowson’s “The Composition of Foods” for the UK [32], and second, if missing in this table, Fineli, the Finnish Food composition table [33] or the Danish Food Composition Table [34]. Individual mono- and disaccharides contents of food items not present in these international tables were imputed using equivalent food items or calculated using recipes included in the NEVO database or manufacturer’s ingredient declarations.

#### 2.2.3. Anthropometric and Other Measurements

Body height (cm) and weight (kg) were measured to the nearest 1 cm and 0.5 kg with the participants wearing light clothing and no shoes (Seca, Hamburg, Germany). Body mass index (BMI) was calculated as kilogram per meter squared (kg/m^2^). Waist and hip circumference, blood pressure, and blood lipid profiles, including triglycerides, total cholesterol, and HDL and LDL cholesterol, were determined as described previously [19]. Finally, smoking status (never, former, or current smoker), total and moderate-to-vigorous physical activity levels (hours/week), medication use, and history of cardiovascular disease (CVD) and cancer (yes/no) were self-reported. Detailed information concerning these measurements can be found in the study protocol of the Maastricht Study [19].

#### 2.2.4. Statistical Methods

All analyses were performed using the software package SPSS Statistics version 23.0 for Windows (SPSS, IBM Corp., Armonk, NY, USA).

Characteristics of the study population were described as means and standard deviations (SD) for continuous variables or as number and proportions of participants per category for categorical variables (% of study population). The Matsuda index was log-transformed to obtain normally distributed data.

Linear regression analyses were performed to assess associations of glucose, fructose and sucrose intake with BCF indices (glucose sensitivity, potentiation factor, C-peptidogenic index, and overall insulin secretion) and the Matsuda index as continuous dependent variables. Logistic regression analyses were performed to assess the associations of mono- and disaccharide intakes with β-cell rate sensitivity, prediabetes, and T2DM. β-cell rate sensitivity was analyzed in tertiles (of which the highest tertile was considered the reference category) as the distribution of β-cell rate sensitivity was positively skewed and could not be normalized by transformations. Associations were presented as standardized regression coefficients (standardized betas (95% CI)) for continuous outcome measures and presented as odds ratios (OR (95% CI)) for categorical outcome measures. 

The independent variables glucose, fructose, and sucrose were analyzed both categorically (comparing tertiles or quintiles) and continuously (per gram increment of glucose, fructose or sucrose intake). To test for linear trends across categories, an ordinal variable with the median value of individual mono- and disaccharide intake for each tertile or quintile was entered in the regression models. 

To adjust for confounding, covariates were included in the regression models if their introduction in the model changed the regression coefficient of mono- and disaccharide intake by >10%. Regardless, model 1 was adjusted for sex and age. Model 2 was additionally adjusted for waist-to-hip-ratio (WHR), education level (low, middle, high), mean arterial blood pressure, CVD, anti-hypertensive medication, lipid-modifying medication, family history of T2DM, moderate-to-vigorous physical activity, and intake of total energy, dietary fibre, and alcohol. In our dataset, smoking status, total serum cholesterol, and the intake of saturated fat and *trans* fat did not confound the associations of the individual mono- and disaccharides intake with BCF, insulin sensitivity, prediabetes, and T2DM, and were therefore not included as covariates. 

To assess BCF relative to the prevailing level of insulin resistance, the Matsuda index was included as a covariate in all regression models with a BCF index as the dependent variable. 

Additional analyses were performed to evaluate the prediabetes subgroups IFG and IGT separately. Furthermore, to evaluate whether the associations of individual mono- and disaccharide intake with glucose metabolism were confounded by total energy intake and WHR, we additionally performed analyses without adjustment for total energy intake and WHR. Finally, analyses with BCF or insulin sensitivity as outcome measure were repeated after exclusion of individuals with previously diagnosed T2DM, as these persons might have adapted their dietary behavior.

## 3. Results

### 3.1. Population Characteristics

Of the 2818 participants included in this study, 1,757 individuals had NGM, 456 had prediabetes, 120 were newly diagnosed with T2DM, and 485 were previously diagnosed with T2DM. The mean (SD) age was 59.7 (8.16) years and 49.5% were male. The prevalence of overweight, obesity, or a high WHR (≥0.80 for women, ≥0.95 for men), hypertension, and history of CVD were lowest among NGM individuals and highest among T2DM individuals. Previously diagnosed T2DM persons had the highest high-density lipoprotein:low-density lipoprotein (HDL:LDL) ratio, most likely due to lipid-modifying medication. BCF and insulin sensitivity were highest among NGM individuals and lowest among T2DM individuals. Moreover, self-reported glucose, fructose, and sucrose intakes were highest in the NGM group. No significant differences in total energy intake were observed between the groups (Table 1).

Individuals with lowest glucose (tertile 1: <12.9 g) and fructose intakes (tertile 1: <14.1 g) were younger, were more often males, included more current smokers, had higher prevalences of overweight/obesity and prediabetes/T2DM, were less physically active, and consumed less fruits and vegetables compared to those individuals with highest intakes of glucose (tertile 3: ≥19.0 g) and fructose (tertile 3: ≥21.2 g). Individuals with the lowest intake of sucrose (tertile 3: <30.0 g) were older, had more often normal weight, were more often males and current smokers, had a higher prevalence of T2DM, and consumed less fruits and vegetables compared to participants that consumed the highest amounts of sucrose (tertile 3: ≥47.9 g).

### 3.2. Associations of Glucose, Fructose, and Sucrose Intake with BCF and Insulin Sensitivity

Glucose intake in the 3rd, 4th, and 5th quintiles were associated with higher β-cell glucose sensitivity, total insulin secretion, and insulin sensitivity, but not with β-cell potentiation factor, β-cell rate sensitivity, or C-peptidogenic index in age- and sex-adjusted models (Table 2 and Table 3). The association between glucose intake and insulin sensitivity remained statistically significant in the fully adjusted model, with a standardized β (95% CI) of 0.07 (0.00, 0.14) for the 5th vs. the 1st quintile (Table 2). Glucose intake was not associated with BCF.

Fructose intake was associated with higher insulin sensitivity for the 5th vs. the 1st quintile in the age- and sex-adjusted model. This association was attenuated in the fully adjusted model with a β of 0.06 (−0.01, 0.13). No associations of fructose intake with BCF were found (Table 2 and Table 3). 

Sucrose intake was associated with higher β-cell glucose sensitivity and total insulin secretion in the age- and sex-adjusted models for the 2nd, 3rd, 4th, and 5th quintiles vs. the 1st quintiles. In the fully adjusted model, the association for the 4th vs. the 1st quintile of sucrose intake with β-cell glucose sensitivity remained significant with a β of 0.09 (0.02, 0.16) (Table 2). Results revealed no significant associations of sucrose intake with other BCF indices and insulin sensitivity.

### 3.3. Associations of Glucose, Fructose, and Sucrose Intake with Prediabetes and T2DM

Glucose intake in the 4th and 5th quintiles were associated with a lower odds of prediabetes in the fully adjusted model with ORs (95% CI) of 0.48 (0.28, 0.81) for quintile 4 vs. quintile 1, and of 0.50 (0.28, 0.90) for quintile 5 vs. quintile 1 (Table 4). 

High fructose intake was associated with lower odds of prediabetes and T2DM as compared to low fructose intake in the age- and sex-adjusted models. These associations did not remain statistically significant in fully adjusted models (Table 4). 

Sucrose intake was not significantly associated with prediabetes or T2DM in any of the models (Table 4).

### 3.4. Additional Analyses

Analysis of associations for subcategories of prediabetes, e.g., IFG (*n* = 123) and IGT (*n* = 333), revealed that higher intake of glucose was associated with a lower odds of IGT, with ORs of 0.51 (0.28, 0.92) for quintile 4 vs. quintile 1, and 0.51 (0.26, 0.99) for quintile 5 vs. quintile 1 in the fully adjusted model (Table S1). Furthermore, when glucose intake was included as a continuous variable, a gram increment of glucose intake was associated with lower odds of both IFG (ORs: 0.94 (0.90, 0.99)) and IGT (OR: 0.97 (0.95, 1.00)) in the fully adjusted models. No significant associations were found for fructose and sucrose intakes with IFG and IGT.

Analyses with the exclusion of all confounders except total energy intake and WHR yielded essentially the same results (Tables S2–S4), except for the association of fructose and insulin sensitivity, which was attenuated after adjustment for WHR (β 0.06 (−0.01, 0.13)).

Exclusion of individuals with previously diagnosed T2DM did not materially affect the results for the primary outcomes BCF and insulin sensitivity (Tables S5 and S6).

## 4. Discussion

This study’s novelty is the evaluation of the associations of glucose, fructose, and sucrose intake with multiple indices of pancreatic BCF and insulin sensitivity. In the fully adjusted models, a positive association of glucose, but not of fructose and sucrose intake, was found with insulin sensitivity. No clear associations were observed for glucose, fructose, and sucrose intake with BCF. 

The positive association of glucose intake with insulin sensitivity has been reported previously [16]. In contrast with the absence of an association between fructose intake and insulin sensitivity in this study, one study [16] showed a positive association, and another study [15] showed an inverse association of fructose intake with insulin sensitivity. Lau et al. [16] did not take dietary fibre intake into account, whereas in our data, adjustment for dietary fibre intake diminished the associations of glucose and fructose intake with insulin sensitivity. A possible explanation for the difference in association between this study and the study of Coello et al. [15] might be the high average fructose intake in their population (mean ± SD: 48.6 ± 31.0 g/day), which might have undermined the health benefits of fruit fibre. Higher intake of sucrose was associated with favourable β-cell glucose sensitivity, but not with insulin sensitivity. The latter finding is in agreement with findings of others [16].

The majority of existing studies focus on the associations of glucose, fructose, and sucrose intake with prediabetes and T2DM. The results of these previous studies are mixed. In the present study, glucose intake was inversely associated with prediabetes. An inverse association of fructose with prediabetes prior to adjustments (Table 3, age- and sex-adjusted model) was observed which proved to be confounded mainly by fibre intake. The absence of an association between fructose and prediabetes in this study contrasts with the positive association observed in another study [4]. However, the positive association observed in that previous study only appeared significant after additional adjustment for dietary fibre [4]. Therefore, it is suggested that dietary fibre may partly account for the association of fructose, derived from fruit and vegetable sources, and glucose metabolism [6,16]. An inverse association of sucrose intake with prediabetes has been reported before [8] but was absent in this study. The absence of an association of glucose, fructose, and sucrose with T2DM found in this study is in agreement with some previous findings [5,7,9,10] but not with all previous studies [2,5,6].

Some previous studies suggest that the beneficial associations of glucose and fructose intake with insulin sensitivity might be confounded by a healthy dietary behaviour, including a high consumption of fibre, fruits, and vegetables [6,35]. Indeed, fruit and vegetables were main sources of glucose and fructose intake in our study. The dietary fibre, vitamins, and antioxidants in fruit and vegetables might improve glucose metabolism [6,36,37,38]. As mentioned before, in this study, the association of glucose intake with insulin sensitivity was attenuated, and the association of fructose intake with insulin sensitivity became nonsignificant after adjusting for dietary fibre. A beneficial role of fructose may also be explained by the relatively low glycaemic response, partly caused by its low glycaemic index, which may result in a lower burden on the β-cells, lower insulin resistance, and a decreased risk of T2DM [39,40]. Another potential explanation for a beneficial association between mono- and disaccharide intake and glucose metabolism is the tendency of obese persons to underreport their mono- and disaccharides consumption [41]. However, previous studies [6,8] and stratified analyses in the current study indicate that the beneficial associations of mono- and disaccharide intake with glucose metabolism were only present in non-obese individuals (BMI < 30 kg/m^2^). 

Other studies suggest that higher intake of glucose, fructose, and sucrose unfavourably affect glucose metabolism through increased energy intake and a subsequent higher WHR [42]. However, as in the case of a previous study [7], this study’s results did not confirm this; the associations between glucose, fructose, and sucrose with BCF, insulin sensitivity, prediabetes, and T2DM were independent of total energy intake and WHR. Regarding glucose intake, the relatively high glycemic index may result in a higher burden on the β-cells, higher insulin resistance and an increased risk of T2DM [5,40]. Finally, concerning fructose intake, it is suggested that the increased fatty acid level causes insulin resistance [42], which results in a higher burden on β-cells [42]. 

Inconsistent findings across studies might be explained by the difference in main sources of mono- and disaccharide intake, as the effects of added vs. naturally occurring sugars might differ [7]. The consumption of mono- and disaccharides through fruit and vegetable intake might be beneficially associated with glucose metabolism, whereas added mono- and disaccharides consumption might be associated with worse glucose metabolism [35]. Furthermore, the number of food items within each product group differs in the FFQs used in individual studies. Hence, the degree by which sources of mono- and disaccharide are measured by FFQs might differ. Regarding the FFQ used in the current study, covered nutrient intake of mono- and disaccharide intake was 98% (Eussen et al. submitted), which reflects an almost complete assessment of the relevant food items. 

One of the strengths of this study is that β-cell dysfunction and insulin resistance provide physiological information on early abnormalities in glucose metabolism that eventually result in prediabetes and T2DM. Furthermore, the continuous outcome measures of BCF and insulin sensitivity prevent individuals to be misclassified as normal or abnormal glucose metabolism. Another strength is the use of multiple indices to assess various aspects of BCF, and the use of non-fasting BCF and insulin sensitivity indices which better reflect metabolic conditions. In addition, the large sample size and the inclusion of participants covering the total glucose metabolism spectrum from NGM to T2DM, and the extensive data collection on demographics and lifestyle factors, allowed a thorough adjustment for confounders. However, a major limitation is the cross-sectional design of our study, which formally rules out causal inference. Nevertheless, all participants completed the FFQ before being informed about their glucose metabolism status, which minimized the risk of reporting bias. Furthermore, previously diagnosed T2DM were excluded in analyses with T2DM as outcome measure, and exclusion of previously diagnosed T2DM in analyses with BCF or insulin sensitivity as outcome measures did not materially affect the results (see Tables S5 and S6). This makes it unlikely that dietary adaptations caused by glucose metabolism status influenced the results. 

Prospective studies will be needed to corroborate these findings. Furthermore, as this is the first study focused on the association of glucose, fructose, and sucrose with non-fasting insulin sensitivity and multiple BCF aspects, more studies are needed to evaluate these potential associations and to investigate the specific mechanisms involved. Finally, because there might be differences in effects of mono- and disaccharide sources on BCF, insulin sensitivity, and glucose metabolism status, future studies should evaluate the associations of added versus natural sources of glucose and fructose intake with BCF, insulin sensitivity, and glucose metabolism status.

## 5. Conclusions

In the current cross-sectional population-based cohort study, high glucose intake was associated with higher insulin sensitivity and a decreased odds of prediabetes, independent of dietary fibre. There is no convincing evidence for associations of glucose, fructose, and sucrose intake with BCF. The predictive effects of mono-and disaccharides on BCF, insulin sensitivity, and T2DM risk still need to be confirmed in future prospective cohort studies.

## Figures and Tables

**Table 1:** Characteristics of study population.

	Total Population	NGM	Prediabetes	ND T2DM	PD T2DM	*p*-Value
	*n* = 2818	*n* = 1757	*n* = 456	*n* = 120	*n* = 485	
Sex (%male)	1395 (49.5)	743 (42.3)	243 (53.3)	75 (62.5)	334 (68.9)	<0.01
Age	59.7 ± 8.16	58.0 ± 8.08	61.7 ± 7.48	63.3 ± 7.49	62.9 ± 7.56	<0.01
BMI category (kg/m^2^)	-	-	-	-	-	<0.01
<25	1048 (37.2)	844 (48.1)	113 (24.8)	20 (16.7)	71 (14.6)	-
25–30	1225 (43.5)	719 (40.9)	224 (49.1)	60 (50.0)	222 (45.8)	-
≥30	544 (19.3)	193 (11.0)	119 (26.1)	40 (33.3)	192 (39.6)	-
WHR cat (%normal)	576 (20.4)	466 (26.5)	68 (14.9)	14 (16.7)	28 (5.8)	<0.01
Systolic blood pressure (mmHg)	134 ± 17.8	131 ± 17.3	137 ± 16.7	143 ± 18.8	141 ± 16.8	<0.01
Diastolic blood pressure (mmHg)	76.3 ± 9.88	75.3 ± 9.94	77.9 ± 9.63	79.2 ± 10.2	77.8 ± 9.31	<0.01
CVD (%yes)	414 (15.1)	209 (12.2)	62 (13.9)	28 (23.5)	115 (24.9)	<0.01
Total PA (hours/week)	14.3 ± 8.07	14.9 ± 8.14	14.2 ± 7.78	13.2 ± 7.49	12.3 ± 7.92	<0.01
MVPA (hours/week)	5.64 ± 4.32	6.13 ± 4.40	5.17 ± 4.06	5.04 ± 4.08	4.33 ± 3.98	<0.01
Total energy intake (kcal)	2192 ± 607	2198 ± 608	2216 ± 586	2129 ± 599	2161 ± 627	0.33
Glucose (g/day)	17.3 ± 9.15	17.9 ± 9.54	16.4 ± 8.03	16.0 ± 7.49	16.2 ± 8.84	<0.01
Fructose (g/day)	19.0 ± 10.1	19.6 ± 10.4	18.2 ± 9.18	17.6 ± 8.35	18.1 ± 10.0	<0.01
Sucrose (g/day)	43.1 ± 24.5	44.3 ± 25.0	42.9 ± 23.6	42.5 ± 26.8	39.3 ± 22.4	<0.01
HbA1c (%)	5.73 ± 0.63	5.45 ± 0.34	5.71 ± 0.40	6.15 ± 0.62	6.68 ± 0.61	<0.01
HDL:LDL ratio	0.54 ± 0.26	0.54 ± 0.27	0.51 ± 0.23	0.48 ± 0.20	0.60 ± 0.29	<0.01
Triglycerides (mmol/L)	1.39 ± 0.82	1.22 ± 0.66	1.61 ± 1.06	1.89 ± 1.07	1.68 ± 0.85	<0.01
Glucose sensitivity (pmol/min/m^2^/mM)	27.6 ± 18.5	33.3 ± 19.3	26.1 ± 12.3	17.8 ± 10.9	11.5 ± 7.97	<0.01
Rate sensitivity (pmol/m^2^/mM)	250 ± 328	307 ± 376	237 ± 223	180 ± 239	81.1 ± 107	<0.01
Potentiation factor	1.63 ± 0.68	1.80 ± 0.73	1.55 ± 0.55	1.38 ± 0.43	1.16 ± 0.34	<0.01
CP_AUC_:G_AUC_ ratio	214 ± 87.8	238 ± 81.8	215 ± 81.0	181 ± 81.5	136 ± 63.0	<0.01
∆CP_30_:G_30_ ratio	469 ± 1141	615 ± 1385	322 ± 683	303 ± 422	142 ± 113	<0.01
Matsuda index	4.04 ± 2.61	4.93 ± 2.65	2.92 ± 1.87	2.25 ± 1.35	2.52 ± 1.82	<0.01

*n*, number; BMI, body mass index; NGM, normal glucose metabolism; ND T2DM, newly diagnosed type 2 diabetes mellitus; PD T2DM, previously diagnosed type 2 diabetes mellitus; CVD, cardiovascular disease; PA, physical activity, MVPA, moderate-to-vigorous physical activity; HbA1C, glycated haemoglobin; HDL, high-density lipoprotein; LDL, low-density lipoprotein.

**Table 2:** Associations of mono- and disaccharide intake with continuous insulin sensitivity and β-cell function (BCF) measures (standardized betas and 95% confidence intervals).

			Continuous	Quintiles Mono- and Disaccharides					
				1 (ref)	2	3	4	5	*P*_trend_
				β	β (95% CI)	β (95% CI)	β (95% CI)	β (95% CI)	
B-cell glucose sensitivity									
	Glucose			<10.19 g	10.19–13.91 g	13.91–17.58 g	17.58–22.79 g	≥22.79 g	-
		1	0.02 (−0.02, 0.05)	0	0.03 (−0.01, 0.08)	0.05 (0, 0.09)	0.05 (0, 0.10)	0.04 (−0.01, 0.09)	0.1
		2	0.01 (−0.05, 0.07)	0	0.03 (−0.03, 0.09)	0.02 (−0.05, 0.08)	0.02 (−0.05, 0.09)	0.06 (−0.02, 0.13)	0.26
	Fructose			<10.86 g	10.86–15.27 g	15.27–19.67 g	19.67–25.51 g	≥25.51 g	-
		1	0 (−0.04, 0.04)	0	0.03 (−0.02, 0.07)	0.04 (−0.01, 0.07)	0.03 (−0.02, 0.08)	0.02 (−0.03, 0.07)	0.44
		2	−0.02 (−0.08, 0.05)	0	−0.01 (−0.08, 0.05)	0.01 (−0.06, 0.07)	−0.02 (−0.09, 0.05)	0.01 (−0.07, 0.09)	1
	Sucrose			<23.36 g	23.36–33.25 g	33.25–43.61 g	43.61–58.74 g	≥58.74 g	-
		1	0.04 (0.01, 0.08)	0	0.06 (0.01, 0.10)	0.06 (0.01, 0.10)	0.07 (0.02, 0.12)	0.06 (0.01, 0.11)	0.05
		2	0.05 (−0.02, 0.12)	0	0.03 (−0.04, 0.09)	0.04 (−0.03, 0.10)	0.09 (0.02, 0.16)	0.05 (−0.03, 0.13)	0.12
B-cell potentiation factor									
	Glucose			<10.19 g	10.19–13.91 g	13.91–17.58 g	17.58–22.79 g	≥22.79 g	-
		1	0 (−0.04, 0.04)	0	−0.02 (−0.07, 0.03)	−0.02 (−0.07, 0.03)	−0.03 (−0.07, 0.02)	−0.01 (−0.06, 0.04)	0.76
		2	0.04 (−0.02, 0.09)	0	0.01 (−0.06, 0.07)	−0.02 (−0.09, 0.05)	0 (−0.07, 0.08)	0.03 (−0.05, 0.11)	0.64
	Fructose			<10.86 g	10.86–15.27 g	15.27–19.67 g	19.67–25.51 g	≥25.51 g	-
		1	0 (−0.04, 0.04)	0	−0.02 (−0.06, 0.03)	−0.02 (−0.07, 0.03)	−0.01 (−0.06, 0.04)	−0.02 (−0.06, 0.03)	0.62
		2	0.05 (−0.02, 0.12)	0	0.01 (−0.06, 0.08)	0 (−0.07, 0.07)	0.02 (−0.05, 0.09)	0.03 (−0.05, 0.11)	0.7
	Sucrose			<23.36 g	23.36–33.25 g	33.25–43.61 g	43.61–58.74 g	≥58.74 g	-
		1	−0.03 (−0.06, 0.01)	0	0.02 (−0.03, 0.07)	0.01 (−0.04, 0.06)	0.03 (−0.02, 0.08)	−0.02 (−0.07, 0.03)	0.36
		2	−0.06 (−0.13, 0.01)	0	0 (−0.07, 0.07)	0.02 (−0.05, 0.09)	0.06 (−0.01, 0.13)	−0.02 (−0.11, 0.06)	1
C-peptidogenic index									
	Glucose			<10.19 g	10.19–13.91 g	13.91–17.58 g	17.58–22.79 g	≥22.79 g	-
		1	0 (−0.04, 0.04)	0	0.03 (−0.02, 0.08)	0.04 (−0.01, 0.09)	−0.01 (−0.06, 0.04)	0.03 (−0.02, 0.08)	0.63
		2	−0.03 (−0.10, 0.05)	0	0.03 (−0.04, 0.10)	0.05 (−0.03, 0.12)	−0.04 (−0.12, 0.04)	0.02 (−0.07, 0.11)	0.8
	Fructose			<10.86 g	10.86–15.27 g	15.27–19.67 g	19.67–25.51 g	≥25.51 g	-
		1	0 (−0.04, 0.04)	0	0.01 (−0.04, 0.06)	0 (−0.05, 0.05)	−0.02 (−0.06, 0.04)	0.02 (−0.03, 0.07)	0.9
		2	−0.02 (−0.09, 0.05)		−0.01 (−0.08, 0.07)	0.01 (−0.07, 0.08)	−0.04 (−0.12, 0.04)	0.01 (−0.08, 0.10)	0.89
	Sucrose			<23.36 g	23.36–33.25 g	33.25–43.61 g	43.61–58.74 g	≥58.74 g	-
		1	0.02 (−0.02, 0.06)	0	0.02 (−0.03, 0.07)	−0.01 (−0.06, 0.04)	0.02 (−0.03, 0.07)	0.02 (−0.03, 0.07)	0.38
		2	−0.01 (−0.08, 0.07)	0	0 (−0.07, 0.08)	−0.05 (−0.12, 0.03)	−0.01 (−0.09, 0.07)	−0.01 (−0.10, 0.09)	0.93
Overall insulin secretion									
	Glucose			<10.19 g	10.19–13.91 g	13.91–17.58 g	17.58–22.79 g	≥22.79 g	-
		1	0.01 (−0.02, 0.05)	0	0.06 (0.02, 0.11)	0.04 (−0.01, 0.09)	0.09 (0.04, 0.13)	0.04 (−0.01, 0.08)	0.19
		2	−0.03 (−0.09, 0.03)	0	0.03 (−0.03, 0.09)	−0.02 (−0.08, 0.05)	0.03 (−0.03, 0.10)	0.01 (−0.07, 0.08)	0.97
	Fructose			<10.86 g	10.86–15.27 g	15.27–19.67 g	19.67–25.51 g	≥25.51 g	-
		1	0 (−0.04, 0.04)	0	0.01 (−0.04, 0.06)	0.02 (−0.03, 0.07)	0.02 (−0.03, 0.07)	0.01 (−0.04, 0.05)	0.78
		2	−0.05 (−0.11, 0.01)	0	−0.03 (−0.09, 0.03)	−0.03 (−0.09, 0.03)	−0.03 (−0.10, 0.03)	−0.03 (−0.10, 0.05)	0.47
	Sucrose			<23.36 g	23.36–33.25 g	33.25–43.61 g	43.61–58.74 g	≥58.74 g	-
		1	0.05 (0.01, 0.09)	0	0.05 (0.01, 0.10)	0.06 (0.01, 0.11)	0.08 (0.03, 0.12)	0.05 (0.01, 0.10)	0.06
		2	−0.01 (−0.07, 0.06)	0	0.01 (−0.06, 0.07)	0.01 (−0.05, 0.07)	0.05 (−0.02, 0.11)	−0.02 (−0.09, 0.06)	0.83
Insulin sensitivity									
	Glucose			<10.19 g	10.19–13.91 g	13.91–17.58 g	17.58–22.79 g	≥22.79 g	-
		1	0.10 (0.07, 0.14)	0	0 (−0.04, 0.05)	0.06 (0.02, 0.11)	0.05 (0, 0.10)	0.10 (0.05, 0.14)	<0.01
		2	0.10 (0.05, 0.16)	0	0.01 (−0.04, 0.07)	0.01 (−0.05, 0.07)	0.04 (−0.02, 0.10)	0.07 (0, 0.14)	0.03
	Fructose			<10.86 g	10.86–15.27 g	15.27–19.67 g	19.67–25.51 g	≥25.51 g	-
		1	0.09 (0.06, 0.13)	0	0.01 (−0.04, 0.06)	0.03 (−0.02, 0.08)	0.04 (−0.01, 0.09)	0.08 (0.04, 0.13)	<0.01
		2	0.08 (0.03, 0.14)	0	0.02 (−0.03, 0.08)	−0.01 (−0.07, 0.05)	0.01 (−0.05, 0.07)	0.06 (−0.01, 0.13)	0.12
	Sucrose			<23.36 g	23.36–33.25 g	33.25–43.61 g	43.61–58.74 g	≥58.74 g	-
		1	0.01 (−0.03, 0.04)	0	0.01 (−0.04, 0.06)	0.04 (−0.01, 0.09)	0.02 (−0.03, 0.07)	0 (−0.05, 0.05)	0.97
		2	0.01 (−0.05, 0.07)	0	0 (−0.05, 0.06)	0.03 (−0.03, 0.09)	0.01 (−0.06, 0.07)	0.03 (−0.04, 0.10)	0.32

CI, confidence interval. Positive values indicate a better β-cell function (BCF) or insulin sensitivity, negative values indicate a lower BCF or insulin sensitivity. M1: sex, age, insulin sensitivity. M2: M1 + waist-to-hip ratio, cardiovascular diseases, blood pressure expressed in mean arterial pressure, lipid-modifying medication, antihypertensive medication, family history of type 2 diabetes mellitus (T2DM), moderate-to-vigorous physical activity, total intake of energy, and dietary fibre and alcohol intake.

**Table 3:** Associations of mono- and disaccharide intake with β-cell rate sensitivity tertiles (odds ratios and 95% confidence intervals).

		Tertile 1 vs. 3 of β-Cell Rate Sensitivity					Tertile 2 vs. 3 of β-Cell Rate Sensitivity				
		Continuous	Tertiles Mono- and Disaccharides				Continuous	Tertiles Mono- and Disaccharides			
			1 (ref)	2	3	*P*_trend_		1 (ref)	2	3	*P*_trend_
			OR	OR (95% CI)	OR (95% CI)			OR	OR (95% CI)	OR (95% CI)	
Glucose			<12.76	12.76–18.99 g	≥18.99 g	-		<12.76	12.76–18.99 g	≥18.99 g	-
	1	1.00 (0.99, 1.01)	1	0.92 (0.73, 1.16)	0.82 (0.65, 1.04)	0.06	1.00 (0.99, 1.01)	1	1.02 (0.81, 1.29)	0.91 (0.72, 1.15)	0.37
	2	1.00 (0.98,1.02)	1	1.03 (0.74, 1.43)	0.87 (0.59, 1.29)	0.47	1.00 (0.98, 1.02)	1	1.09 (0.79, 1.50)	0.80 (0.55, 1.18)	0.24
Fructose			<13.94 g	13.94–21.17 g	≥21.17 g	-		<13.94 g	13.94–21.17 g	≥21.17 g	-
	1	1.00 (0.99, 1.01)	1	0.86 (0.68, 1.08)	0.86 (0.68, 1.09)	0.18	1.00 (0.99, 1.01)	1	1.03 (0.82, 1.30)	0.90 (0.71, 1.14)	0.34
	2	1.00 (0.98, 1.02)	1	0.92 (0.66, 1.29)	0.86 (0.58, 1.27)	0.42	1.00 (0.99, 1.02)	1	1.08 (0.78, 1.48)	0.83 (0.57, 1.21)	0.24
Sucrose			<29.98	29.98–47.64 g	≥47.64 g	-		<29.98	29.98–47.64 g	≥47.64 g	-
	1	1.00 (1.00, 1.00)	1	0.79 (0.63, 1.00)	0.90 (0.71, 1.15)	0.38	1.00 (1.00, 1.00)	1	0.87 (0.70, 1.10)	0.88 (0.69, 1.12)	0.30
	2	1.00 (0.99, 1.01)	1	0.97 (0.69, 1.35)	1.00 (0.66, 1.50)	0.45	1.00 (1.00, 1.01)	1	0.97 (0.70, 1.34)	1.07 (0.72, 1.59)	0.86

CI, confidence interval. Third tertile of β-cell rate sensitivity is the reference group (best rate sensitivity). Values <1.00 indicate a better β-cell rate sensitivity, values >1.00 indicate a lower β-cell rate sensitivity. M1: sex, age, insulin sensitivity. M2: M1 + waist-to-hip-ratio, cardiovascular diseases, blood pressure expressed in mean arterial pressure, lipid-modifying medication, antihypertensive medication, family history of T2DM, moderate-to-vigorous physical activity, total intake of energy, and dietary fibre and alcohol intake.

**Table 4:** Associations of mono- and disaccharide intake with prediabetes and T2DM (odds ratio and 95% confidence intervals).

		**Continuous**	**Quintiles Mono- and Disaccharides**					
			**1 (ref)**	**2**	**3**	**4**	**5**	***P*_trend_**
			**OR**	**OR (95% CI)**	**OR (95% CI)**	**OR (95% CI)**	**OR (95% CI)**	
Prediabetes								
Glucose			<10.61 g	10.61–14.43 g	14.43–18.16 g	18.16–23.66 g	≥23.66 g	-
	1	0.98 (0.96, 0.99)	1	0.95 (0.69, 1.30)	0.78 (0.56, 1.08)	0.58 (0.41, 0.81)	0.61 (0.44, 0.85)	<0.01
	2	0.97 (0.94, 0.99)	1	0.78 (0.50, 1.22)	0.85 (0.53, 1.37)	0.48 (0.28, 0.81)	0.50 (0.28, 0.90)	<0.01
Fructose			<11.14 g	11.14–15.72 g	15.72–20.11 g	20.11–26.43 g	≥26.43 g	-
	1	0.98 (0.97, 0.99)	1	1.02 (0.74, 1.41)	0.97 (0.70, 1.33)	0.63 (0.45, 0.90)	0.71 (0.51, 0.99)	<0.01
	2	0.98 (0.96, 1.00)	1	1.02 (0.64, 1.63)	1.10 (0.68, 1.77)	0.82 (0.48, 1.40)	0.83 (0.46, 1.48)	0.29
Sucrose			<24.53 g	24.53–34.50 g	34.50–45.30 g	45.30–61.58 g	≥61.58 g	-
	1	1.00 (0.99, 1.00)	1	0.87 (0.63, 1.21)	0.94 (0.68, 1.30)	0.78 (0.56, 1.10)	0.86 (0.62, 1.21)	0.33
	2	1.00 (0.99, 1.01)	1	1.00 (0.63, 1.59)	0.87 (0.54, 1.42)	0.68 (0.41, 1.15)	0.71 (0.40, 1.26)	0.13
		**Continuous**	**Tertiles mono- and disaccharides**					
			**1 (ref)**	**2**	**3**			***P*_trend_**
			**OR**	**OR (95% CI)**	**OR (95% CI)**			
T2DM								
Glucose			<13.10 g	13.10–19.49 g	≥19.49 g			-
	1	0.98 (0.95, 1.00)	1	0.97 (0.62, 1.51)	0.75 (0.47, 1.19)			0.18
	2	0.98 (0.93, 1.02)	1	1.72 (0.87, 3.37)	1.05 (0.44, 2.52)			0.87
Fructose			<14.34 g	14.34–21.64 g	≥21.64 g			-
	1	0.98 (0.96, 1.00)	1	1.01 (0.65, 1.54)	0.61 (0.37, 0.99)			0.06
	2	0.97 (0.94, 1.01)	1	1.35 (0.70, 2.59)	0.54 (0.22 (2.86)			0.43
Sucrose			<31.10 g	31.10–49.36 g	≥49.36 g			-
	1	1.00 (0.99, 1.01)	1	0.84 (0.54, 1.32)	0.81 (0.51, 1.28)			0.37
	2	1.01 (0.99, 1.02)	1	1.26 (0.64, 2.48)	1.00 (0.43, 2.31)			0.98

CI, confidence interval. Values <1.00 indicate a lower odds of prediabetes or T2DM, values >1.00 indicate a higher odds of prediabetes or T2DM. M1: sex, age. M2: M1 + waist-to-hip ratio, cardiovascular diseases, blood pressure expressed in mean arterial pressure, lipid-modifying medication, antihypertensive medication, family history of T2DM, moderate-to-vigorous physical activity, total intake of energy, dietary fibre and alcohol intake.

## Supplementary Materials

The following are available online at http://www.mdpi.com/2072-6643/9/4/380/s1, Table S1: Associations of mono- and disaccharides with IFG and IGT, Table S2: Associations of mono- and disaccharides with BCF and insulin sensitivity, with and without adjustment for total EI and WHR, Table S3: Associations of mono- and disaccharides with β-cell rate sensitivity, with and without adjustment for total EI and WHR, Table S4: Associations of mono- and disaccharides with prediabetes and T2DM, with and without adjustment for total EI and WHR, Table S5: Associations of mono- and disaccharides with BCF and insulin sensitivity, with exclusion of persons with PD T2DM, Table S6: Associations of mono- and disaccharides with β-cell rate sensitivity, with exclusion of persons with PD T2DM.
