# Supplementary material for: Associations of Dietary Glucose, Fructose, and Sucrose with β-Cell Function, Insulin Sensitivity, and Type 2 Diabetes in the Maastricht Study
Source: Nutrients. 2017 Apr 13;9(4):380. doi: 10.3390/nu9040380 (PMC5409719; doi:10.3390/nu9040380)
Supplement: Supplementary file 1 [file nutrients-09-00380-s001.docx]

**Table S1.** Associations of mono- and disaccharides with IFG and IGT.

|  | | **Continuous** | **Quintiles Mono- and Disaccharides** | | | | | |
| --- | --- | --- | --- | --- | --- | --- | --- | --- |
|  |  |  | **1 (ref)** | **2** | **3** | **4** | **5** | ***P*_trend_** |
|  |  |  | **OR** | **OR (95%CI)** | **OR (95%CI)** | **OR (95%CI)** | **OR (95%CI)** |  |
| IFG | | | | | | | | |
| Glucose |  |  | <10.69 g | 10.69–14.49 g | 14.49–18.08 g | 18.08–23.36 g | >23.36 g | - |
|  | 1 | 0.98 (0.95, 1.00) | 1 | 1.62 (0.94, 2.77) | 1.14 (0.64, 2.02) | 0.59 (0.30, 1.14) | 0.77 (0.42, 1.43) | 0.04 |
|  | 2 | 0.94 (0.90, 0.99) | 1 | 1.31 (0.56, 3.05) | 41.12 (0.45, 2.87) | 0.50 (0.17, 1.47) | 0.45 (0.13, 1.51) | 0.07 |
| Fructose |  |  | <11.18 g | 11.18–15.70 g | 15.70–20.15 g | 20.15–26.18 g | >26.18 g | - |
|  | 1 | 0.98 (0.96, 1.00) | 1 | 1.16 (0.68, 1.99) | 0.92 (0.53, 1.62) | 0.41 (0.21, 0.83) | 0.90 (0.51, 1.58) | 0.20 |
|  | 2 | 0.97 (0.94, 1.01) | 1 | 0.93 (0.37, 2.30) | 1.15 (0.47, 2.85) | 0.39 (0.12, 1.27) | 0.97 (0.34, 2.84) | 0.72 |
| Sucrose |  |  | <24.50 g | 24.50–34.26 g | 34.26–45.03 g | 45.03–60.00 g | >60.00 g | - |
|  | 1 | 1.00 (0.99, 1.00) | 1 | 0.93 (0.52, 1.67) | 0.76 (0.42, 1.38) | 0.78 (0.44, 1.41) | 0.77 (0.43, 1.37) | 0.36 |
|  | 2 | 0.99 (0.97, 1.00) | 1 | 1.14 (0.47, 2.78) | 0.60 (0.23, 1.61) | 0.62 (0.23, 1.68) | 0.30 (0.09, 1.00) | 0.02 |
| IGT | | | | | | | | |
| Glucose |  |  | <10.53 g | 10.53–14.37 g | 14.37–18.03 g | 18.03–23.23 g | >23.23 g | - |
|  | 1 | 0.98 (0.96, 0.99) | 1 | 0.80 (0.56, 1.14) | 0.71 (0.49, 1.02) | 0.59 (0.41, 0.86) | 0.54 (0.37, 0.79) | 0.07 |
|  | 2 | 0.97 (0.95, 1.00) | 1 | 0.75 (0.46, 1,23) | 0.82 (0.48, 1.40) | 0.51 (0.28, 0.92) | 0.51 (0.26, 0.99) | 0.06 |
| Fructose |  |  | <11.06 g | 11.06–15.69 g | 15.69–20.00 g | 20.00–25.94 g | >25.94 g | - |
|  | 1 | 0.98 (0.97, 0.99) | 1 | 0.90 (0.62, 1.29) | 0.96 (0.67, 1.39) | 0.69 (0.47, 1.02) | 0.60 (0.41, 0.89) | 0.23 |
|  | 2 | 0.98 (0.96, 1.01) | 1 | 0.94 (0.56, 1.58) | 1.08 (0.64, 1.84) | 0.89 (0.50, 1.61) | 0.70 (0.36, 1.34) | 0.78 |
| Sucrose |  |  | <24.22 g | 24.22–34.13 g | 34.13–44.43 g | 44.43–59.66 g | >59.66 g | - |
|  | 1 | 1.00 (0.99, 1.00) | 1 | 0.82 (0.57, 1.19) | 0.97 (0.68, 1.40) | 0.79 (0.54, 1.15) | 0.86 (0.59, 1.26) | 0.37 |
|  | 2 | 1.00 (0.99, 1.01) | 1 | 0.93 (0.56, 1.56) | 0.96 (0.57, 1.64) | 0.70 (0.39, 1.24) | 0.86 (0.46, 1.62) | 0.03 |

CI, confidence interval; IFG, impaired fasting glucose; IGT, impaired glucose tolerance. Values <1.00 indicate a lower odds of IFG or IGT, values >1.00 indicate a higher odds of IFG or IGT. M1: sex, age. M2: M1 + waist-to-hip ratio, cardiovascular diseases, blood pressure expressed in mean arterial pressure, lipid-modifying medication, antihypertensive medication, family history of T2DM, moderate-to-vigorous physical activity, total intake of energy, dietary fibre and alcohol intake.

**Table S2.** Associations of mono- and disaccharides with BCF and insulin sensitivity, with and without adjustment for total EI and WHR.

|  | | | **Continuous** | **Quintiles Mono- and Disaccharides** | | | | | |
| --- | --- | --- | --- | --- | --- | --- | --- | --- | --- |
|  |  |  |  | **1 (ref)** | **2** | **3** | **4** | **5** | ***P*_trend_** |
|  |  |  |  | **β** | **β (95%CI)** | **β (95%CI)** | **β (95%CI)** | **β (95%CI)** |  |
| B–cell glucose sensitivity | |  |  |  |  |  |  |  |  |
|  | Glucose |  |  | <10.19 g | 10.19–13.91 g | 13.91–17.58 g | 17.58–22.79 g | >22.79 g |  |
|  |  | 1 | 0.02 (–0.02, 0.05) | 0 | 0.03 (–0.01, 0.08) | 0.05 (0.00, 0.09) | 0.05 (0.00, 0.10) | 0.04 (–0.01, 0.09) | 0.10 |
|  |  | 2 | 0.01 (–0.05, 0.08) | 0 | 0.03 (–0.03, 0.09) | 0.02 (–0.05, 0.08) | 0.03 (–0.04, 0.10) | 0.06 (–0.02, 0.14) | 0.20 |
|  |  | 3 | 0.01 (–0.05, 0.16) | 0 | 0.03 (–0.03, 0.09) | 0.02 (–0.05, 0.08) | 0.03 (–0.05, 0.09) | 0.06 (–0.02, 0.14) | 0.23 |
|  |  | 4 | 0.01 (–0.05, 0.07) | 0 | 0.03 (–0.03, 0.09) | 0.02 (–0.05, 0.08) | 0.02 (–0.05, 0.09) | 0.06 (–0.02, 0.13) | 0.26 |
|  | Fructose |  |  | <10.86 g | 10.86–15.27 g | 15.27–19.67 g | 19.67–25.51 g | >25.51 g |  |
|  |  | 1 | 0.00 (–0.04, 0.04) | 0 | 0.03 (–0.02, 0.07) | 0.04 (–0.01, 0.07) | 0.03 (–0.02, 0.08) | 0.02 (–0.03, 0.07) | 0.44 |
|  |  | 2 | –0.01 (–0.08, 0.05) | 0 | –0.01 (–0.08, 0.05) | 0.01 (–0.06, 0.07) | –0.02 (–0.09, 0.05) | 0.01 (–0.07, 0.09) | 0.92 |
|  |  | 3 | –0.01 (–0.08, 0.05) | 0 | –0.01 (–0.08, 0.05) | 0.01 (–0.06, 0.07) | –0.02 (–0.09, 0.05) | 0.01 (–0.07, 0.09) | 0.95 |
|  |  | 4 | –0.02 (–0.08, 0.05) | 0 | –0.01 (–0.08, 0.05) | 0.01 (–0.06, 0.07) | –0.02 (–0.09, 0.05) | 0.01 (–0.07, 0.09) | 1.00 |
|  | Sucrose |  |  | <23.36 g | 23.36–33.25 g | 33.25–43.61 g | 43.61–58.74 g | >58.74 g |  |
|  |  | 1 | 0.04 (0.01, 0.08) | 0 | 0.06 (0.01, 0.10) | 0.06 (0.01, 0.10) | 0.07 (0.02, 0.12) | 0.06 (0.01, 0.11) | 0.05 |
|  |  | 2 | 0.04 (–0.01, 0.09) | 0 | 0.03 (–0.04, 0.09) | 0.04 (–0.03, 0.10) | 0.09 (0.03, 0.16) | 0.05 (–0.02, 0.11) | 0.09 |
|  |  | 3 | 0.05 (–0.02, 0.12) | 0 | 0.03 (–0.04, 0.09) | 0.04 (–0.03, 0.10) | 0.09 (0.02, 0.16) | 0.05 (–0.03, 0.13) | 0.10 |
|  |  | 4 | 0.05 (–0.02, 0.12) | 0 | 0.03 (–0.03, 0.09) | 0.04 (–0.03, 0.10) | 0.10 (0.03, 0.17) | 0.06 (–0.02, 0.13) | 0.12 |
| B–cell potentiation factor | |  |  |  |  |  |  |  |  |
|  | Glucose |  |  | <10.19 g | 10.19–13.91 g | 13.91–17.58 g | 17.58–22.79 g | >22.79 g |  |
|  |  | 1 | 0.00 (–0.04, 0.04) | 0 | –0.02 (–0.07, 0.03) | –0.02 (–0.07, 0.03) | –0.03 (–0.07, 0.02) | –0.01 (–0.06, 0.04) | 0.76 |
|  |  | 2 | 0.05 (–0.02, 0.11) | 0 | 0.00 (–0.06, 0.07) | –0.02 (–0.09, 0.05) | 0.00 (–0.07, 0.08) | 0.03 (–0.05, 0.12) | 0.56 |
|  |  | 3 | 0.05 (–0.02, 0.12) | 0 | 0.01 (–0.06, 0.07) | –0.02 (–0.08, 0.06) | 0.01 (–0.06, 0.08) | 0.04 (–0.04, 0.12) | 0.52 |
|  |  | 4 | 0.04 (–0.02, 0.09) | 0 | 0.01 (–0.06, 0.07) | –0.02 (–0.09, 0.05) | 0.00 (–0.07, 0.08) | 0.03 (–0.05, 0.11) | 0.64 |
|  | Fructose |  |  | <10.86 g | 10.86–15.27 g | 15.27–19.67 g | 19.67–25.51 g | >25.51 g |  |
|  |  | 1 | 0.00 (–0.04, 0.04) | 0 | –0.02 (–0.06, 0.03) | –0.02 (–0.07, 0.03) | –0.01 (–0.06, 0.04) | –0.02 (–0.06, 0.03) | 0.62 |
|  |  | 2 | 0.05 (–0.01, 0.12) | 0 | 0.01 (–0.06, 0.07) | 0.01 (–0.06, 0.08) | 0.02 (–0.05, 0.09) | 0.03 (–0.05, 0.11) | 0.60 |
|  |  | 3 | 0.06 (–0.01, 0.12) | 0 | 0.01 (–0.06, 0.08) | 0.01 (–0.06, 0.08) | 0.02 (–0.05, 0.10) | 0.03 (–0.05, 0.12) | 059 |
|  |  | 4 | 0.05 (–0.02, 0.12) | 0 | 0.01 (–0.06, 0.08) | 0.00 (–0.07, 0.07) | 0.02 (–0.05, 0.09) | 0.03 (–0.05, 0.11) | 0.70 |
|  | Sucrose |  |  | <23.36 g | 23.36–33.25 g | 33.25–43.61 g | 43.61–58.74 g | >58.74 g |  |
|  |  | 1 | –0.03 (–0.06, 0.01) | 0 | 0.02 (–0.03, 0.07) | 0.01 (–0.04, 0.06) | 0.03 (–0.02, 0.08) | –0.02 (–0.07, 0.03) | 0.36 |
|  |  | 2 | –0.05 (–0.10, 0.01) | 0 | 0.00 (–0.07, 0.07) | 0.02 (–0.05, 0.09) | 0.06 (–0.01, 0.13) | –0.03 (–0.10, 0.04) | 0.97 |
|  |  | 3 | –0.05 (–0.12, 0.02) | 0 | 0.00 (–0.07, 0.07) | 0.02 (–0.05, 0.09) | 0.07 (–0.01, 0.14) | –0.02 (–0.10, 0.07) | 0.83 |
|  |  | 4 | –0.06 (–0.13, 0.01) | 0 | 0.00 (–0.07, 0.07) | 0.02 (–0.05, 0.09) | 0.06 (–0.01, 0.13) | –0.02 (–0.11, 0.06) | 1.00 |
| C–peptidogenic index | |  |  |  |  |  |  |  |  |
|  | Glucose |  |  | <10.19 g | 10.19–13.91 g | 13.91–17.58 g | 17.58–22.79 g | >22.79 g |  |
|  |  | 1 | 0.00 (–0.04, 0.04) | 0 | 0.03 (–0.02, 0.08) | 0.04 (–0.01, 0.09) | –0.01 (–0.06, 0.04) | 0.03 (–0.02, 0.08) | 0.63 |
|  |  | 2 | –0.03 (–0.10, 0.05) | 0 | 0.03 (–0.04, 0.10) | 0.05 (–0.03, 0.12) | –0.04 (–0.12, 0.04) | 0.02 (–0.08, 0.11) | 0.81 |
|  |  | 3 | –0.03 (–0.10, 0.05) | 0 | 0.03 (–0.04, 0.10) | 0.05 (–0.03, 0.12) | –0.04 (–0.12, 0.04) | 0.02 (–0.07, 0.11) | 0.89 |
|  |  | 4 | –0.03 (–0.10, 0.05) | 0 | 0.03 (–0.04, 0.10) | 0.05 (–0.03, 0.12) | –0.04 (–0.12, 0.04) | 0.02 (–0.07, 0.11) | 0.90 |
|  | Fructose |  |  | <10.86 g | 10.86–15.27 g | 15.27–19.67 g | 19.67–25.51 g | >25.51 g |  |
|  |  | 1 | 0.00 (–0.04, 0.04) | 0 | 0.01 (–0.04, 0.06) | 0.00 (–0.05, 0.05) | –0.02 (–0.06, 0.04) | 0.02 (–0.03, 0.07) | 0.65 |
|  |  | 2 | –0.02 (–0.10, 0.05) | 0 | –0.01 (–0.08, 0.07) | 0.01 (–0.07, 0.08) | –0.04 (–0.12, 0.04) | 0.01 (–0.09, 0.09) | 0.86 |
|  |  | 3 | –0.02 (–0.10, 0.05) | 0 | –0.01 (–0.08, 0.07) | 0.01 (–0.07, 0.08) | –0.04 (–0.12, 0.04) | 0.01 (–0.08, 0.10) | 0.89 |
|  |  | 4 | –0.02 (–0.09, 0.05) | 0 | –0.01 (–0.08, 0.07) | 0.01 (–0.07, 0.08) | –0.04 (–0.12, 0.04) | 0.01 (–0.08, 0.10) | 0.89 |
|  | Sucrose |  |  | <23.36 g | 23.36–33.25 g | 33.25–43.61 g | 43.61–58.74 g | >58.74 g |  |
|  |  | 1 | 0.02 (–0.02, 0.06) | 0 | 0.02 (–0.03, 0.07) | –0.01 (–0.06, 0.04) | 0.02 (–0.03, 0.07) | 0.02 (–0.03, 0.07) | 0.38 |
|  |  | 2 | –0.01 (–0.07, 0.05) | 0 | 0.00 (–0.07, 0.07) | –0.05 (–0.13, 0.02) | –0.02 (–0.09, 0.06) | –0.02 (–0.09, 0.06) | 063 |
|  |  | 3 | –0.01 (–0.08, 0.07) | 0 | 0.00 (–0.07, 0.08) | –0.05 (–0.12, 0.03) | –0.01 (–0.09, 0.07) | –0.01 (–0.10, 0.08) | 0.92 |
|  |  | 4 | –0.01 (–0.08, 0.07) | 0 | 0.00 (–0.07, 0.08) | –0.05 (–0.12, 0.03) | –0.01 (–0.09, 0.07) | –0.01 (–0.10, 0.09) | 0.93 |
| Overall insulin secretion | |  |  |  |  |  |  |  |  |
|  | Glucose |  |  | <10.19 g | 10.19–13.91 g | 13.91–17.58 g | 17.58–22.79 g | >22.79 g |  |
|  |  | 1 | 0.01 (–0.02, 0.05) | 0 | 0.06 (0.02, 0.11) | 0.04 (–0.01, 0.09) | 0.09 (0.04, 0.13) | 0.04 (–0.01, 0.08) | 0.19 |
|  |  | 2 | –0.03 (–0.09, 0.03) | 0 | 0.03 (–0.03, 0.09) | –0.02 (–0.08, 0.05) | 0.03 (–0.03, 0.10) | 0.01 (–0.06, 0.08) | 0.89 |
|  |  | 3 | –0.03 (–0.09, 0.03) | 0 | 0.03 (–0.03, 0.09) | –0.02 (–0.08, 0.05) | 0.03 (–0.03, 0.10) | 0.01 (–0.07, 0.08) | 1.00 |
|  |  | 4 | –0.03 (–0.09, 0.03) | 0 | 0.03 (–0.03, 0.09) | –0.02 (–0.08, 0.05) | 0.03 (–0.03, 0.10) | 0.01 (–0.07, 0.08) | 0.97 |
|  | Fructose |  |  | <10.86 g | 10.86–15.27 g | 15.27–19.67 g | 19.67–25.51 g | >25.51 g |  |
|  |  | 1 | 0.00 (–0.04, 0.04) | 0 | 0.01 (–0.04, 0.06) | 0.02 (–0.03, 0.07) | 0.02 (–0.03, 0.07) | 0.01 (–0.04, 0.05) | 0.78 |
|  |  | 2 | –0.05 (–0.11, 0.01) | 0 | –0.03 (–0.09, 0.03) | –0.03 (–0.09, 0.03) | –0.03 (–0.10, 0.03) | –0.03 (–0.10, 0.05) | 0.49 |
|  |  | 3 | –0.05 (–0.11, 0.01) | 0 | –0.03 (–0.09, 0.03) | –0.03 (–0.09, 0.03) | –0.03 (–0.10, 0.03) | –0.03 (–0.10, 0.05) | 0.45 |
|  |  | 4 | –0.05 (–0.11, 0.01) | 0 | –0.03 (–0.09, 0.03) | –0.03 (–0.09, 0.03) | –0.03 (–0.10, 0.03) | –0.03 (–0.10, 0.05) | 0.47 |
|  | Sucrose |  |  | <23.36 g | 23.36–33.25 g | 33.25–43.61 g | 43.61–58.74 g | >58.74 g |  |
|  |  | 1 | 0.05 (0.01, 0.09) | 0 | 0.05 (0.01, 0.10) | 0.06 (0.01, 0.11) | 0.08 (0.03, 0.12) | 0.05 (0.01, 0.10) | 0.06 |
|  |  | 2 | 0.01 (–0.04, 0.06) | 0 | 0.01 (–0.06, 0.07) | 0.02 (–0.05, 0.08) | 0.05 (–0.01, 0.12) | 0.00 (–0.07, 0.06) | 0.79 |
|  |  | 3 | –0.01 (–0.07, 0.06) | 0 | 0.01 (–0.06, 0.07) | 0.01 (–0.05, 0.07) | 0.04 (–0.02, 0.11) | –0.02 (–0.10, 0.06) | 0.79 |
|  |  | 4 | –0.01 (–0.07, 0.06) | 0 | 0.01 (–0.06, 0.07) | 0.01 (–0.05, 0.07) | 0.05 (–0.02, 0.11) | –0.02 (–0.09, 0.06) | 0.83 |
| Insulin sensitivity | |  |  |  |  |  |  |  |  |
|  | Glucose |  |  | <10.19 g | 10.19–13.91 g | 13.91–17.58 g | 17.58–22.79 g | >22.79 g |  |
|  |  | 1 | 0.10 (0.07, 0.14) | 0 | 0.00 (–0.04, 0.05) | 0.06 (0.02, 0.11) | 0.05 (0.00, 0.10) | 0.10 (0.05, 0.14) | <0.01 |
|  |  | 2 | 0.12 (0.06, 0.18) | 0 | 0.01 (–0.05, 0.06) | 0.02 (–0.04, 0.08) | 0.04 (–0.02, 0.11) | 0.09 (0.02, 0.16) | 0.01 |
|  |  | 3 | 0.13 (0.07, 0.19) | 0 | 0.01 (–0.05, 0.07) | 0.02 (–0.04, 0.09) | 0.06 (–0.01, 0.12) | 0.10 (0.03, 0.17) | <0.01 |
|  |  | 4 | 0.10 (0.05, 0.16) | 0 | 0.01 (–0.04, 0.07) | 0.01 (–0.05, 0.07) | 0.04 (–0.02, 0.10) | 0.07 (0.00, 0.14) | 0.03 |
|  | Fructose |  |  | <10.86 g | 10.86–15.27 g | 15.27–19.67 g | 19.67–25.51 g | >25.51 g |  |
|  |  | 1 | 0.09 (0.06, 0.13) | 0 | 0.01 (–0.04, 0.06) | 0.03 (–0.02, 0.08) | 0.04 (–0.01, 0.09) | 0.08 (0.04, 0.13) | <0.01 |
|  |  | 2 | 0.10 (0.04, 0.16) | 0 | 0.02 (–0.04, 0.08) | 0.00 (–0.06, 0.06) | 0.02 (–0.04, 0.09) | 0.08 (0.01, 0.16) | 0.04 |
|  |  | 3 | 0.10 (0.04, 0.16) | 0 | 0.02 (–0.04, 0.08) | 0.01 (–0.06, 0.06) | 0.03 (–0.04, 0.09) | 0.09 (0.02, 0.16) | 0.03 |
|  |  | 4 | 0.08 (0.03, 0.14) | 0 | 0.02 (–0.03, 0.08) | –0.01 (–0.07, 0.05) | 0.01 (–0.05, 0.07) | 0.06 (–0.01, 0.13) | 0.12 |
|  | Sucrose |  |  | <23.36 g | 23.36–33.25 g | 33.25–43.61 g | 43.61–58.74 g | >58.74 g |  |
|  |  | 1 | 0.01 (–0.03, 0.04) | 0 | 0.01 (–0.04, 0.06) | 0.04 (–0.01, 0.09) | 0.02 (–0.03, 0.07) | 0.00 (–0.05, 0.05) | 0.97 |
|  |  | 2 | –0.03 (–0.08, 0.02) | 0 | –0.01 (–0.07, 0.05) | 0.03 (–0.03, 0.09) | 0.00 (–0.07, 0.06) | –0.01 (–0.07, 0.06) | 0.93 |
|  |  | 3 | 0.03 (–0.03, 0.09) | 0 | 0.00 (–0.05, 0.07) | 0.05 (–0.02, 0.11) | 0.03 (–0.03, 0.10) | 0.06 (–0.01, 0.13) | 0.07 |
|  |  | 4 | 0.01 (–0.05, 0.07) | 0 | 0.00 (–0.05, 0.06) | 0.03 (–0.03, 0.09) | 0.01 (–0.06, 0.07) | 0.03 (–0.04, 0.10) | 0.32 |

CI, confidence interval; IFG, impaired fasting glucose; IGT, impaired glucose tolerance. Values <1.00 indicate a lower odds of IFG or IGT, values >1.00 indicate a higher odds of IFG or IGT. M1: sex, age. M2: M1 + waist-to-hip ratio, cardiovascular diseases, blood pressure expressed in mean arterial pressure, lipid-modifying medication, antihypertensive medication, family history of T2DM, moderate-to-vigorous physical activity, total intake of energy, dietary fibre and alcohol intake.

**Table S3.** Associations of mono- and disaccharides with β-cell rate sensitivity, with and without adjustment for total EI and WHR.

|  | | **Tertile 1 vs. 3 of β-Cell Rate Sensitivity** | | | | | **Tertile 2 vs. 3 of β-Cell Rate Sensitivity** | | | | |
| --- | --- | --- | --- | --- | --- | --- | --- | --- | --- | --- | --- |
|  |  | **Continuous** | **Tertiles Mono- and Disaccharides** | | | | **Continuous** | **Tertiles Mono- and Disaccharides** | | | |
|  |  |  | **1 (ref)** | **2** | **3** | ***P*_trend_** |  | **1 (ref)** | **2** | **3** | ***P*_trend_** |
|  |  |  | **OR** | **OR (95% CI)** | **OR (95% CI)** |  |  | **OR** | **OR (95% CI)** | **OR (95% CI)** |  |
| Glucose |  |  |  |  |  |  |  |  |  |  |  |
|  | 1 | 1.00 (0.99, 1.01) | 1 | 0.92 (0.73, 1.16) | 0.82 (0.65, 1.04) | 0.06 | 1.00 (0.99, 1.01) | 1 | 1.02 (0.81, 1.29) | 0.91 (0.72, 1.15) | 0.37 |
|  | 2 | 1.00 (0.98,1.02) | 1 | 1.04 (0.75, 1.45) | 0.89 (0.60, 1.31) | 0.52 | 1.00 (0.98,1.02) | 1 | 1.09 (0.79, 1.49) | 0.80 (0.55, 1.18) | 0.24 |
| Fructose |  | 1.00 (0.98,1.02) | 1 | 1.03 (0.74, 1.43) | 0.87 (0.59, 1.28) | 0.47 | 1.00 (0.98,1.02) | 1 | 1.09 (0.79, 1.50) | 0.81 (0.55, 1.19) | 0.25 |
|  | 1 | 1.00 (0.98,1.02) | 1 | 1.03 (0.74, 1.43) | 0.87 (0.59, 1.29) | 0.47 | 1.00 (0.98, 1.02) | 1 | 1.09 (0.79, 1.50) | 0.80 (0.55, 1.18) | 0.24 |
|  | 2 |  | <13.94 g | 13.94–21.17 g | >21.17 g | - | - | <13.94 g | 13.94–21.17 g | >21.17 g | - |
| Sucrose |  | 1.00 (0.99, 1.01) | 1 | 0.86 (0.68, 1.08) | 0.86 (0.68, 1.09) | 0.18 | 1.00 (0.99, 1.01) | 1 | 1.03 (0.82, 1.30) | 0.90 (0.71, 1.14) | 0.34 |
|  | 1 | 1.00 (0.98, 1.02) | 1 | 0.92 (0.66, 1.29) | 0.87 (0.59, 1.28) | 0.43 | 1.00 (0.99, 1.02) | 1 | 1.08 (0.78, 1.49) | 0.83 (0.57, 1.21) | 0.24 |
|  | 2 | 1.00 (0.98, 1.01) | 1 | 0.92 (0.66, 1.29) | 0.86 (0.58, 1.26) | 0.41 | 1.00 (0.99, 1.02) | 1 | 1.08 (0.78, 1.49) | 0.83 (0.57, 1.22) | 0.25 |

CI, confidence interval. Third tertile of rate sensitivity is the reference group (best rate sensitivity). Values <1.00 indicate a better β-cell rate sensitivity, values >1.00 indicate a lower β-cell rate sensitivity. M1: sex, age, insulin sensitivity. M2: M1 + cardiovascular diseases, blood pressure expressed in mean arterial pressure, lipid-modifying medication, antihypertensive medication, family history of T2DM, moderate-to-vigorous physical activity, dietary fibre intake and alcohol intake. M3: M2 + total energy intake. M4: M3 + waist-to-hip ratio.

**Table S4.** Associations of mono- and disaccharides with prediabetes and T2DM, with and without adjustment for total EI and WHR.

|  | | **Continuous** | **Quintiles Mono- and Disaccharides** | | | | | |
| --- | --- | --- | --- | --- | --- | --- | --- | --- |
|  |  |  | **1 (ref)** | **2** | **3** | **4** | **5** | ***P*_trend_** |
|  |  |  | **OR** | **OR (95%CI)** | **OR (95%CI)** | **OR (95%CI)** | **OR (95%CI)** |  |
| Prediabetes | | | | | | | | |
| Glucose |  |  | <10.61 g | 10.61–14.43 g | 14.43–18.16 g | 18.16–23.66 g | >23.66 g | - |
|  | 1 | 0.98 (0.96, 0.99) | 1 | 0.95 (0.69, 1.30) | 0.78 (0.56, 1.08) | 0.58 (0.41, 0.81) | 0.61 (0.44, 0.85) | <0.01 |
|  | 2 |  | 1 | 0.78 (0.50, 1,22) | 0.85 (0.53, 1.37) | 0.48 (0.28, 0.81) | 0.50 (0.28, 0.90) | <0.01 |
|  | 3 |  | 1 | 0.78 (0.50, 1,22) | 0.85 (0.53, 1.37) | 0.48 (0.28, 0.81) | 0.50 (0.28, 0.90) | <0.01 |
|  | 4 | 0.97 (0.94, 0.99) | 1 | 0.78 (0.50, 1,22) | 0.85 (0.53, 1.37) | 0.48 (0.28, 0.81) | 0.50 (0.28, 0.90) | <0.01 |
| Fructose |  |  | <11.14 g | 11.14–15.72 g | 15.72–20.11 g | 20.11–26.43 g | >26.43 g | - |
|  | 1 | 0.98 (0.97, 0.99) | 1 | 1.02 (0.74, 1.41) | 0.97 (0.70, 1.33) | 0.63 (0.45, 0.90) | 0.71 (0.51, 0.99) | <0.01 |
|  | 2 |  | 1 | 1.02 (0.64, 1.63) | 1.10 (0.68, 1.77) | 0.82 (0.48, 1.40) | 0.83 (0.46, 1.48) | 0.17 |
|  | 3 |  | 1 | 1.02 (0.64, 1.63) | 1.10 (0.68, 1.77) | 0.82 (0.48, 1.40) | 0.83 (0.46, 1.48) | 0.15 |
|  | 4 | 0.98 (0.96, 1.00) | 1 | 1.02 (0.64, 1.63) | 1.10 (0.68, 1.77) | 0.82 (0.48, 1.40) | 0.83 (0.46, 1.48) | 0.29 |
| Sucrose |  |  | <24.53 g | 24.53–34.50 g | 34.50–45.30 g | 45.30–61.58 g | >61.58 g | - |
|  | 1 | 1.00 (0.99, 1.00) | 1 | 0.87 (0.63, 1.21) | 0.94 (0.68, 1.30) | 0.78 (0.56, 1.10) | 0.86 (0.62, 1.21) | 0.33 |
|  | 2 |  | 1 | 1.00 (0.63, 1.59) | 0.87 (0.54, 1.42) | 0.68 (0.41, 1.15) | 0.71 (0.40, 1.26) | 0.31 |
|  | 3 |  | 1 | 1.00 (0.63, 1.59) | 0.87 (0.54, 1.42) | 0.68 (0.41, 1.15) | 0.71 (0.40, 1.26) | 0.07 |
|  | 4 | 1.00 (0.99, 1.01) | 1 | 1.00 (0.63, 1.59) | 0.87 (0.54, 1.42) | 0.68 (0.41, 1.15) | 0.71 (0.40, 1.26) | 0.13 |
|  | | **Continuous** | **Tertiles mono– and disaccharides** | | | | | |
|  |  |  | **1 (ref)** | **2** | **3** |  |  | ***P*_trend_** |
|  |  |  | **OR** | **OR (95%CI)** | **OR (95%CI)** |  |  |  |
| T2DM | | | | | | | | |
| Glucose |  |  | <13.10 g | 13.10–19.49 g | >19.49 g |  |  | - |
|  | 1 | 0.98 (0.95, 1.00) | 1 | 0.97 (0.62, 1.51) | 0.75 (0.47, 1.19) |  |  | 0.18 |
|  | 2 | 0.97 (0.92, 1.01) | 1 | 1.56 (0.81, 3.03) | 0.85 (0.36, 2.00) |  |  | 0.75 |
|  | 3 | 0.97 (0.93, 1.01) | 1 | 1.60 (0.83, 3.12) | 0.91 (0.38, 2.17) |  |  | 0.89 |
|  | 4 | 0.98 (0.93, 1.02) | 1 | 1.72 (0.87, 3.37) | 1.05 (0.44, 2.52) |  |  | 0.87 |
| Fructose |  |  | <14.34 g | 14.34–21.64 g | >21.64 g |  |  | - |
|  | 1 | 0.98 (0.96, 1.00) | 1 | 1.01 (0.65, 1.54) | 0.61 (0.37, 0.99) |  |  | 0.06 |
|  | 2 | 0.97 (0.93, 1.01) | 1 | 1.26 (0.66, 2.40) | 0.46 (0.19 (1.15) |  |  | 0.25 |
|  | 3 | 0.97 (0.93, 1.01) | 1 | 1.26 (0.66, 2.41) | 0.48 (0.19 (1.19) |  |  | 0.28 |
|  | 4 | 0.97 (0.94, 1.01) | 1 | 1.35 (0.70, 2.59) | 0.54 (0.22 (2.86) |  |  | 0.43 |
| Sucrose |  |  | <31.10 g | 31.10–49.36 g | >49.36 g |  |  | - |
|  | 1 | 1.00 (0.99, 1.01) | 1 | 0.84 (0.54, 1.32) | 0.81 (0.51, 1.28) |  |  | 0.37 |
|  | 2 | 1.00 (0.99, 1.01) | 1 | 1.11 (0.58, 2.12) | 0.75 (0.36, 1.57) |  |  | 0.41 |
|  | 3 | 1.00 (0.99, 1.02) | 1 | 1.17 (0.60, 2.28) | 0.87 (0.38, 2.02) |  |  | 0.73 |
|  | 4 | 1.01 (0.99, 1.02) | 1 | 1.26 (0.64, 2.48) | 1.00 (0.43, 2.31) |  |  | 0.98 |

CI, confidence interval. Values <1.00 indicate a lower odds of prediabetes or T2DM, values >1.00 indicate a higher odds of prediabetes or T2DM. M1: sex, age, insulin sensitivity. M2: M1 + cardiovascular diseases, blood pressure expressed in mean arterial pressure, lipid-modifying medication, antihypertensive medication, family history of T2DM, moderate-to-vigorous physical activity, dietary fibre intake and alcohol intake. M3: M2 + total energy intake. M4: M3 + waist-to-hip ratio.

**Table S5.** Associations of mono- and disaccharides with BCF and insulin sensitivity, with exclusion of persons with PD T2DM.

|  | | | **Continuous** | **Quintiles Mono- and Disaccharides** | | | | | |
| --- | --- | --- | --- | --- | --- | --- | --- | --- | --- |
|  |  |  |  | 1 (ref) | 2 | 3 | 4 | 5 | *P*_trend_ |
|  |  |  |  | β | β (95%CI) | β (95%CI) | β (95%CI) | β (95%CI) |  |
| B-cell glucose sensitivity | |  |  |  |  |  |  |  |  |
|  | Glucose |  |  | <10.19 g | 10.19–13.91 g | 13.91–17.58 g | 17.58–22.79 g | >22.79 g |  |
|  |  | 1 | –0.01 (–0.05, 0.04) | 0 | 0.03 (–0.02, 0.08) | 0.04 (–0.01, 0.10) | 0.02 (–0.03, 0.10) | 0.03 (–0.03, 0.08) | 0.47 |
|  |  | 2 | 0.02 (–0.05, 0.09) | 0 | 0.02 (–0.05, 0.09) | –0.01 (–0.09, 0.06) | 0.01 (–0.07, 0.08) | 0.05 (–0.04, 0.14) | 0.30 |
|  | Fructose |  |  | <10.86 g | 10.86–15.27 g | 15.27–19.67 g | 19.67–25.51 g | >25.51 g |  |
|  |  | 1 | –0.01 (–0.05, 0.03) | 0 | 0.03 (–0.03, 0.08) | 0.04 (–0.01, 0.09) | 0.02 (–0.04, 0.07) | 0.01 (–0.05, 0.06) | 0.96 |
|  |  | 2 | –0.01 (–0.08, 0.06) | 0 | 0.00 (–0.07, 0.07) | 0.02 (–0.05, 0.09) | –0.02 (–0.10, 0.06) | 0.00 (–0.09, 0.09) | 0.82 |
|  | Sucrose |  |  | <23.36 g | 23.36–33.25 g | 33.25–43.61 g | 43.61–58.74 g | >58.74 g |  |
|  |  | 1 | 0.02 (–0.02, 0.06) | 0 | 0.03 (–0.02, 0.08) | 0.04 (–0.01, 0.09) | 0.05 (0.00, 0.10) | 0.03 (–0.02, 0.09) | 0.31 |
|  |  | 2 | 0.04 (–0.03, 0.12) | 0 | 0.03 (–0.04, 0.10) | 0.04 (–0.03, 0.12) | 0.10 (0.02, 0.17) | 0.06 (–0.03, 0.15) | 0.13 |
| B-cell potentiation factor | |  |  |  |  |  |  |  |  |
|  | Glucose |  |  | <10.19 g | 10.19–13.91 g | 13.91–17.58 g | 17.58–22.79 g | >22.79 g |  |
|  |  | 1 | –0.02 (–0.06, 0.02) | 0 | –0.03 (–0.08, 0.03) | –0.03 (–0.09, 0.02) | –0.06 (–0.12, –0.01) | –0.04 (–0.09, 0.02) | 0.14 |
|  |  | 2 | 0.04 (–0.04, 0.12) | 0 | 0.01 (–0.06, 0.08) | –0.01 (–0.09, 0.07) | –0.01 (–0.09, 0.07) | 0.02 (–0.07, 0.11) | 0.97 |
|  | Fructose |  |  | <10.86 g | 10.86–15.27 g | 15.27–19.67 g | 19.67–25.51 g | >25.51 g |  |
|  |  | 1 | –0.02 (–0.06, 0.02) | 0 | –0.02 (–0.07, 0.03) | –0.03 (–0.08, 0.03) | –0.03 (–0.08, 0.03) | –0.04 (–0.10, 0.01) | 0.13 |
|  |  | 2 | 0.05 (–0.03, 0.12) | 0 | 0.03 (–0.05, 0.10) | 0.01 (–0.06, 0.09) | 0.02 (–0.06, 0.10) | 0.02 (–0.07, 0.11) | 0.94 |
|  | Sucrose |  |  | <23.36 g | 23.36–33.25 g | 33.25–43.61 g | 43.61–58.74 g | >58.74 g |  |
|  |  | 1 | –0.06 (–0.11, –0.02) | 0 | –0.02 (–0.07, 0.03) | –0.02 (–0.07, 0.03) | 0.00 (–0.06, 0.05) | –0.08 (–0.14, –0.03) | <0.01 |
|  |  | 2 | –0.10 (–0.17, –0.02) | 0 | –0.02 (–0.09, 0.06) | –0.01 (–0.09, 0.06) | 0.04 (–0.04, 0.12) | –0.08 (–0.17, 0.01) | 0.30 |
| C-peptidogenic index | |  |  |  |  |  |  |  |  |
|  | Glucose |  |  | <10.19 g | 10.19–13.91 g | 13.91–17.58 g | 17.58–22.79 g | >22.79 g |  |
|  |  | 1 | –0.01 (–0.05, 0.04) | 0 | 0.04 (–0.02, 0.09) | 0.04 (–0.02, 0.09) | –0.03 (–0.08, 0.03) | 0.03 (–0.03, 0.08) | 0.83 |
|  |  | 2 | –0.03 (–0.12, 0.05) | 0 | 0.03 (–0.05, 0.11) | 0.04 (–0.04, 0.12) | –0.05 (–0.14, 0.04) | 0.01 (–0.09, 0.11) | 0.76 |
|  | Fructose |  |  | <10.86 g | 10.86–15.27 g | 15.27–19.67 g | 19.67–25.51 g | >25.51 g |  |
|  |  | 1 | –0.01 (–0.05, 0.04) | 0 | 0.01 (–0.05, 0.06) | 0.00 (–0.05, 0.06) | –0.02 (–0.08, 0.03) | 0.02 (–0.04, 0.07) | 0.78 |
|  |  | 2 | –0.03 (–0.11, 0.06) |  | –0.01 (–0.09, 0.07) | 0.02 (–0.07, 0.10) | –0.04 (–0.13, 0.04) | 0.00 (–0.10, 0.10) | 0.83 |
|  | Sucrose |  |  | <23.36 g | 23.36–33.25 g | 33.25–43.61 g | 43.61–58.74 g | >58.74 g |  |
|  |  | 1 | 0.01 (–0.03, 0.05) | 0 | 0.00 (–0.06, 0.05) | –0.02 (–0.08, 0.03) | 0.01 (–0.05, 0.06) | 0.01 (–0.05, 0.06) | 0.66 |
|  |  | 2 | –0.01 (–0.10, 0.07) | 0 | –0.01 (–0.09, 0.07) | –0.06 (–0.14, 0.02) | –0.02 (–0.11, 0.07) | –0.03 (–0.13, 0.07) | 0.67 |
| Overall insulin secretion | |  |  |  |  |  |  |  |  |
|  | Glucose |  |  | <10.19 g | 10.19–13.91 g | 13.91–17.58 g | 17.58–22.79 g | >22.79 g |  |
|  |  | 1 | –0.01 (–0.05, 0.03) | 0 | 0.07 (0.02, 0.12) | 0.03 (0.02, 0.12) | 0.06 (0.01, 0.12) | 0.02 (–0.03, 0.07) | 0.85 |
|  |  | 2 | –0.03 (–0.10, 0.04) | 0 | 0.02 (–0.05, 0.08) | –0.07 (–0.14, 0.00) | 0.01 (–0.06, 0.08) | –0.02 (–0.10, 0.06) | 0.56 |
|  | Fructose |  |  | <10.86 g | 10.86–15.27 g | 15.27–19.67 g | 19.67–25.51 g | >25.51 g |  |
|  |  | 1 | –0.01 (–0.05, 0.03) | 0 | 0.03 (–0.02, 0.08) | 0.00 (–0.05, 0.05) | 0.00 (–0.05, 0.05) | 0.00 (–0.05, 0.05) | 0.58 |
|  |  | 2 | –0.05 (–0.11, 0.02) | 0 | –0.02 (–0.09, 0.05) | –0.06 (–0.12, 0.01) | –0.05 (–0.12, 0.02) | –0.04 (–0.12, 0.04) | 0.26 |
|  | Sucrose |  |  | <23.36 g | 23.36–33.25 g | 33.25–43.61 g | 43.61–58.74 g | >58.74 g |  |
|  |  | 1 | 0.02 (–0.02, 0.06) | 0 | 0.02 (–0.03, 0.08) | 0.02 (–0.03, 0.07) | 0.05 (0.00, 0.10) | 0.01 (0.04, 0.06) | 0.61 |
|  |  | 2 | –0.03 (–0.10, 0.04) | 0 | 0.02 (–0.05, 0.09) | –0.01 (–0.07, 0.06) | 0.05 (–0.03, 0.12) | –0.03 (–0.11, 0.05) | 0.57 |
| Insulin sensitivity | |  |  |  |  |  |  |  |  |
|  | Glucose |  |  | <10.19 g | 10.19–13.91 g | 13.91–17.58 g | 17.58–22.79 g | >22.79 g |  |
|  |  | 1 | 0.11 (0.07, 0.15) | 0 | 0.01 (–0.05, 0.06) | 0.04 (–0.01, 0.10) | 0.05 (–0.01, 0.10) | 0.11 (0.05, 0.16) | <0.01 |
|  |  | 2 | 0.13 (0.07, 0.19) | 0 | 0.02 (–0.04, 0.08) | 0.02 (–0.05, 0.08) | 0.04 (–0.02, 0.11) | 0.10 (0.02, 0.17) | 0.01 |
|  | Fructose |  |  | <10.86 g | 10.86–15.27 g | 15.27–19.67 g | 19.67–25.51 g | >25.51 g |  |
|  |  | 1 | 0.10 (0.06, 0.14) | 0 | 0.00 (–0.05, 0.05) | 0.02 (–0.03, 0.07) | 0.05 (0.00, 0.10) | 0.09 (0.03, 0.14) | <0.01 |
|  |  | 2 | 0.11 (0.04, 0.17) | 0 | 0.02 (–0.05, 0.08) | 0.00 (–0.07, 0.06) | 0.00 (–0.07, 0.07) | 0.07 (0.00, 0.15) | 0.07 |
|  | Sucrose |  |  | <23.36 g | 23.36–33.25 g | 33.25–43.61 g | 43.61–58.74 g | >58.74 g |  |
|  |  | 1 | 0.01 (–0.03, 0.05) | 0 | 0.01 (–0.04, 0.07) | 0.03 (–0.02, 0.09) | 0.02 (–0.03, 0.07) | 0.01 (–0.04, 0.06) | 0.80 |
|  |  | 2 | 0.02 (–0.05, 0.08) | 0 | 0.00 (–0.06, 0.06) | 0.02 (–0.05, 0.08) | 0.01 (–0.05, 0.08) | 0.04 (–0.04, 0.12) | 0.24 |

PD T2DM, previously diagnosed T2DM; CI, confidence interval. Positive values indicate a better BCF or insulin sensitivity, negative values indicate a lower BCF or insulin sensitivity. M1: sex, age, insulin sensitivity. M2: M1 + WHR, cardiovascular diseases, blood pressure expressed in mean arterial pressure, lipid-modifying medication, antihypertensive medication, family history of T2DM, moderate-to-vigorous physical activity, total intake of energy, dietary fibre and alcohol intake.

**Table S6.** Associations of mono- and disaccharides with β-cell rate sensitivity, with exclusion of persons with PD T2DM.

|  | **Tertile 1 vs. 3 of β-Cell Rate Sensitivity** | | | | | | **Tertile 2 vs. 3 of β-Cell Rate Sensitivity** | | | | |
| --- | --- | --- | --- | --- | --- | --- | --- | --- | --- | --- | --- |
|  | | **Continuous** | **Tertiles Mono- and Disaccharides** | | | | **Continuous** | **Tertiles Mono- and Disaccharides** | | | |
|  |  |  | **1 (ref)** | **2** | **3** | ***P*_trend_** |  | **1 (ref)** | **2** | **3** | ***P*_trend_** |
|  |  |  | **OR** | **OR (95%CI)** | **OR (95%CI)** |  |  | **OR** | **OR (95%CI)** | **OR (95%CI)** |  |
| Glucose |  |  | <12.76 | 12.76–18.99 g | >18.99 g |  |  | <12.76 | 12.76–18.99 g | >18.99 g |  |
|  | 1 | 1.00 (0.99, 1.01) | 1 | 0.92 (0.73, 1.16) | 0.82 (0.65, 1.04) | 0.69 | 1.00 (0.98, 1.02) | 1 | 1.02 (0.81, 1.29) | 0.91 (0.72, 1.15) | 0.50 |
|  | 2 | 1.00 (0.98,1.02) | 1 | 1.03 (0.74, 1.43) | 0.87 (0.59, 1.29) | 0.98 | 1.00 (0.98, 1.02) | 1 | 1.09 (0.79, 1.50) | 0.80 (0.55, 1.18) | 0.38 |
| Fructose |  |  | <13.94 g | 13.94–21.17 g | >21.17 g |  |  | <13.94 g | 13.94–21.17 g | >21.17 g |  |
|  | 1 | 1.00 (0.99, 1.01) | 1 | 0.86 (0.68, 1.08) | 0.86 (0.68, 1.09) | 0.46 | 1.00 (0.99, 1.01) | 1 | 1.03 (0.82, 1.30) | 0.90 (0.71, 1.14) | 0.55 |
|  | 2 | 1.00 (0.98, 1.02) | 1 | 0.92 (0.66, 1.29) | 0.86 (0.58, 1.27) | 0.55 | 1.01 (0.99, 1.02) | 1 | 1.08 (0.78, 1.48) | 0.83 (0.57, 1.21) | 0.31 |
| Sucrose |  |  | <29.98 | 29.98–47.64 g | >47.64 g |  |  | <29.98 | 29.98–47.64 g | >47.64 g |  |
|  | 1 | 1.00 (1.00, 1.01) | 1 | 0.79 (0.63, 1.00) | 0.90 (0.71, 1.15) | 0.97 | 1.00 (1.00, 1.01) | 1 | 0.87 (0.70, 1.10) | 0.88 (0.69, 1.12) | 0.75 |
|  | 2 | 1.00 (1.00, 1.01) | 1 | 0.97 (0.69, 1.35) | 1.00 (0.66, 1.50) | 0.86 | 1.00 (1.00, 1.01) | 1 | 0.97 (0.70, 1.34) | 1.07 (0.72, 1.59) | 0.88 |

PD T2DM, previously diagnosed T2DM; CI, confidence interval. Positive values indicate a better BCF or insulin sensitivity, negative values indicate a lower BCF or insulin sensitivity. M1: sex, age, insulin sensitivity. M2: M1 + WHR, cardiovascular diseases, blood pressure expressed in mean arterial pressure, lipid-modifying medication, antihypertensive medication, family history of T2DM, moderate-to-vigorous physical activity, total intake of energy, dietary fibre and alcohol intake.
